# *PTPN11* mutation with additional somatic alteration indicates unfavorable outcome in juvenile myelomonocytic leukemia: a retrospective clinical study from a single center

**DOI:** 10.1007/s00431-019-03468-8

**Published:** 2019-12-05

**Authors:** Yan Miao, Benshang Li, Lixia Ding, Hua Zhu, Changying Luo, Jianmin Wang, Chengjuan Luo, Jing Chen

**Affiliations:** Department of Hematology and Oncology, Shanghai Children’s Medical Center, School of Medicine, Shanghai Jiao Tong University, Dongfang Road 1678, Shanghai, 200127 China

**Keywords:** Gene mutation, Juvenile myelomonocytic leukemia, *PTPN11*, Secondary mutation

## Abstract

Juvenile myelomonocytic leukemia (JMML) is a heterogeneous childhood leukemia. The management of patients with JMML requires accurate assessment of genetic and clinical features to help in patient risk stratification. This study aimed to investigate the association between genomic alterations and prognosis in children with JMML. Genomic DNA was extracted from a total of 93 patients with JMML for targeted sequencing. Univariable and multivariable analysis were used to evaluate the correlation between gene mutations and prognosis of the patients. Patients with *PTPN11* mutation exhibited significantly lower event-free survival (EFS) compared with non-*PTPN11* mutations (*P* = 0.005). Patients without or with one somatic alteration at diagnosis showed significantly better prognosis in comparison with those with more than two alterations (*P* = 0.009). *PTPN11* mutation with additional alterations showed significantly the poorest outcome in comparison with those with only one non-*PTPN11* mutation, only one *PTPN11* mutation, and combined mutations without *PTPN11*, respectively (*P* < 0.0001).

*Conclusion*: Both *PTPN11* mutation and the number of somatic alterations detected at diagnosis are likely to be the major determinant of outcome in JMML. The subgroup of patients with *PTPN11* mutation showed the shortest survival which was even worsened when a secondary mutation was present.

## Introduction

Juvenile myelomonocytic leukemia (JMML) is a rare, early childhood neoplasm with features characteristic of both myelodysplastic and myeloproliferative disorders. Most JMML cases are severe, with the only curative treatment being hematopoietic stem cell transplantation (HSCT) [1, 4]. However, clinical evolution is heterogeneous, with occasional transformation to acute myeloid leukemia (AML) and frequent relapses after HSCT, whereas some rare “long-term survivors” experience spontaneous remission and survive without treatment [3, 4, 16]. The management of patients with JMML requires accurate assessment of genetic and clinical features to help in patient risk stratification [2, 5]. We hypothesized that complete genomic characterization of JMML would aid in distinguishing these cases.

Driver mutations converge on the RAS-signaling pathway. Mutations in *NF1*, *NRAS*, *KRAS*, *PTPN11*, and *CBL* currently allow for a molecular diagnosis in 85% of patients [14, 15, 23]. Recently, secondary mutations, such as *SETBP1* and *JAK3*, were identified in a number of patients with JMML at diagnosis [20]. We therefore sequenced a series of genes involved in signal transduction, splicing, transcription, and epigenetic modification, in addition to the canonical RAS genes. These findings uncovered a level of genetic complexity in JMML.

## Methods

### Patient samples

This study included 93 patients with JMML, diagnosed from 2009 to 2018 at the Department of Hematology and Oncology, Shanghai Children’s Medical Center (SCMC), China. All patients harboring gene alterations were diagnosed according to WHO criteria for the diagnosis of JMML.

### Targeted NGS

Genomic DNA was extracted from total peripheral blood (PB) in 68 cases or bone marrow (BM) aspirates in 25 cases at diagnosis, and 500  ng of DNA were fragmented on the Covaris M220 Focused-ultrasonicator and purified to yield fragments of 450–550 bp. Fragmented DNA was modified by end-repair, 3′ end adenylylation and Illumina adaptor ligation. The adapter-ligated DNA fragments were captured by a customized panel of biotinylated oligoprobes (Roche NimbleGen) and amplified. Amplified material was validated and quantified using an Agilent 2100 Bioanalyzer. Each DNA library was then sequenced on an Illumina X Ten for paired-end reads at 150 bp by the targeted gene panel designed (Table 1), which was designed to cover the RAS-signaling gene regions and the possible synergistic gene regions involved in signal transduction, splicing, transcription, and epigenetic modification. Genetic analysis in non-hematopoietic tissues (such as fingernails, hair follicles, or buccal swab samples) of patients or PB of the patients’ parents was of critical importance in addition to the screening in leukemic cells to determine whether the mutation was somatic or germline, considering the milder clinical course described in germline mutations. When potential leukocyte contamination in hair follicles or buccal swab samples existed, fingernails or the parents’ samples were done.

**Table 1:** Genes involved in the panel screening the samples with JMML

RAS-signaling Gene	Signal transduction	Transcription factor	Epigenetic modifier	Splicesome
*CBL*	*SETBP1*	*GATA2*	*ASXL1*	*SRSF2*
*NF1*	*NOTCH1*	*RUNX1*	*EZH2*	*ZRSR2*
*NRAS*	*JAK2*	*CEBPA*	*DNMT3A*	*U2AF1*
*KRAS*	*JAK3*	*ETV6*	*FLT3*	*SF3B1*
*PTPN11*	*CSF3R*	*FOXN1*	*KDM6A*	
*HRAS*	*ARHGAP26*	*TERT*		
*NF2*	*IDH1*	*TERC*		
	*MLLT11*	*TET2*		
	*MPL*	*TP53*		
	*NPM1*	*SH2B3*		
	*PDGFRB*			
	*RABEP1*			
	*ROBO1*			
	*ROBO2*			
	*HEPACAM2*			

### Bioinformatics

The raw FASTQ data were trimmed, filtering the low quality or undefined bases, and deleting the reads that has a length less than 50 bp. The clean reads were then aligned to the hg19 reference genome using the BWA-MEM (BWA-0.7.10). Duplicates were removed using Picard. BAM files were further processed according to Genome Analysis Toolkit best practices (https://www.broadinstitute.org/gatk/guide/bp_step.php?p=1), by performing Indel Realignment and Base Quality Recalibration. Variants were called using VarScan 2 and the variants were annotated using ANNOVAR. The final output positive mutations were confirmed by using the Sanger sequencing method.

### Statistical analysis

Patients’ outcome data updated on December 31, 2018, were used. Event-free survival (EFS) time was calculated from diagnosis to the first failure, including death, relapse, or treatment abandonment due to disease progression. Overall survival (OS) time was considered from the time of diagnosis to death. When no events occurred, the observation was censored at the time of last follow-up. EFS and OS curves were estimated by the Kaplan-Meier method and compared with the log-rank test. Univariable and multivariable analyses were performed using the Cox model. The chi-square test was used to assess the associations and distribution characteristics between categorical variables. All *P* values were 2-sided, with values of < 0.05 indicating statistical significance. For statistical analysis, we used the SPSS Mac23.0.0 (IBM Corp.) software package.

## Results

### Clinical and biological features at diagnosis

In the 93 cases, the median age of diagnosis was 32.6 (range, 3.3–168.0) months, 50 cases were less than 24 months, 61 cases were male, and 32 were female. White blood cells’ number at diagnosis was 29.2 (2.7–127.1) × 10^3^/μL, platelet count was 73.3 (4.0–357.0) × 10^3^/μL, and monocytes’ number was 5.2 (1.1–48.1) × 10^3^/μL. Percentage of myeloid and erythroid precursors on PB smear was 5.0 (0–24.0)%. Median percentage of BM blasts was 7.8 (0.4–20.0)%.

There were 30 cases with *PTPN11* mutation, 20 with *NF1* mutation, 16 with *NRAS* mutation, 10 with *KRAS* mutation, 4 with *CBL* mutation, and 13 with other mutations unable to be simply categorized to the five classical RAS-signaling mutations aforementioned (Table 2). In these cases, there were 89 somatic mutations and only 4 germline mutations, including 2 *NF1*, 1 *PTPN11*, and 1 *KRAS* mutation.

**Table 2:** Characteristics of patients with JMML

Variable	Value	Total cases (*N*)
Gender (male/female)	61/32	93
Median age at diagnosis (months)	32.6 (3.3–168.0)	93
Median white blood cells at diagnosis × 10^3^/μL (range)	29.2 (2.7–127.1)	92
Median count of hemoglobin	92.1 (30.0–143.0)	92
Median monocyte at diagnosis × 10^3^/μL (range)	5.2 (1.0–48.1)	92
Median platelet count at diagnosis × 10^3^/μL (range)	73.3 (4.0–357.0)	92
Myeloid or erythroid precursors on PB smear (%)	5.0 (0–24.0)	81
Median percentage of BM blasts at diagnosis (%)	7.8 (0.4–20.0)	83
Median percentage of HbF at diagnosis	21.0 (1.5–64.50)	44
Lactic dehydrogenase (U/L)	936.0 (280.0–4200.0)	43
Monosomy 7 (*N*)	12 (20.7%)	58
Abnormal karyotype (*N*)	21 (33.8%)	62
HSCT (*N*)	45 (48.4%)	93
RAS-signaling genes (*N*)		93
*PTPN11*	30 (32.3%)	
*NF1*	20 (21.5%)	
*NRAS*	16 (17.2%)	
*KRAS*	10 (10.8%)	
*CBL*	4 (4.3%)	
Other	13 (13.9%)	
Germline or somatic mutations		93
Germline	4/93 (4.30%)	
Somatic	89/93 (95.7%)	
Number of somatic alterations at diagnosis (*N*)		93
0 or 1	61 (65.6%)	
2 or more	32 (34.4%)	
Mutation subtypes stratified by *PTPN11* status and alteration number (*N*)		93
Only one non-*PTPN11* mutation	41 (44.1%)	
Only one *PTPN11* mutation	14 (15.1%)	
Combined mutations without *PTPN11*	20 (21.5%)	
*PTPN11* mutation with additional alterations	18 (19.3%)	

After diagnosis, we recommended swift HSCT for all children with *NF1*, somatic *PTPN11*, or *KRAS* mutations, and for the vast majority of children with somatic *NRAS* mutations. Children with *CBL* mutations were followed closely and were not offered HSCT immediately. Transplantation was considered, however, if disease progressed. Yet some patients did not receive HSCT due to fee or some other reasons. Forty-five patients received transplantation in 2–3 months after diagnosis. Thirty-two patients received mild chemotherapy before transplantation, with 15 receiving hydroxycarbamide, 8 receiving 6-mercaptopurine, 3 receiving 13-cis retinoic acid, and 6 receiving combined chemotherapy of these drugs. And 4 patients received courses of intensive chemotherapy for AML, 2 with daunorubicin-cytarabine-etoposide, and 2 with homoharringtonine-cytarabine. Five patients received decitabine before HSCT. The other 48 patients did not receive HSCT. Among these cases, only 2 patients of these cases received recorded chemotherapy, 1 with 13-cis retinoic acid, and 1 with a course of daunorubicin-cytarabine-etoposide before death.

After a median follow-up of 25 (0–125) months, 51 children were alive; By the Kaplan-Meier method, the 5 -year

OS was estimated at 50.7%. In the untransplanted patients, 9 cases died of primary disease due to lack of treatment and 1 died of transformation to AML. In the HSCT patients,7 cases died of relapse, 4 died of transplantation complications, and 2 died of AML. Relapse remained the major cause of death in JMML after HSCT. Some other studies have assigned poorer prognostic significance to several clinical and laboratory characteristics in patients with JMML, such as age > 24 months, male sex, lower platelet count, and monosomy 7 [12, 13]. However, in our cohort, no clinical characteristics, including age, sex, platelet count, higher hemoglobin F (HbF) concentration for age, abnormal G-band karyotype of chromosome, and monosomy 7 detected by fluorescence in situ hybridization, reached significance in univariate Cox analysis (Table 3).

**Table 3:** Univariate analysis of the EFS analysis in childhood JMML

Variable	*N*/total	EFS (%)	HR (95% CI)	*P*
Gender				
Female	32/93	48.4	1	
Male	61/93	44.6	1.15 (0.62–2.11)	0.659
Age at diagnosis				
≤ 24 months	50/93	53.5	1	
> 24 months	43/93	34.2	1.43 (0.81–2.54)	0.217
Platelet count at diagnosis (× 10^3^/μL)				
≥ 40	59/92	45.7	1	
< 40	33/92	43.8	1.30 (0.72–2.33)	0.389
Myeloid or erythroid precursors on PB smear				
No	17/81	37.2	1.01 (0.54–1.87)	
Yes	64/81	48.0	0.99 (0.46–2.16)	0.987
HbF at diagnosis				
Not elevated for age	7/45	71.4	1	
Elevated for age	38/45	46.9	2.28 (0.53–9.83)	0.27
Monosomy 7				
Negative	47/59	43.9	1	
Positive	12/59	65.6	0.51 (0.17–1.45)	0.208
Karyotype				
Normal	41/62	48.5	1	
Abnormal	21/62	40.8	1.09(0.54–2.25)	0. 796
Germline or somatic mutation				
Germline	4/93	66.7	1	
Somatic	89/93	44.0	1.53 (0.21–11.15)	0.675
*NF1* status				
Mutation absent	67/93	45.0	1	
Mutation present	26/93	43.0	1.01 (0.54–1.87)	0.988
*PTPN11* status				
Mutation absent	55/93	57.4	1	
Mutation present	38/93	27.2	2.25 (1.27–3.99)	*0.005*
Somatic alterations at diagnosis				
0 or 1	61/93	56.4	1	
2 or more	32/93	25.0	2.13 (1.21–3.76)	*0.009*
HSCT				
Yes	45/93	54.1	1	
No	48/93	33.6	1.95 (1.09–3.47)	*0.024*

Clinical and laboratory characteristics of 93 cases, including sex, age, platelet count, myeloid or erythroid precursors on PB smear, HbF concentration, monosomy 7, abnormal karyotype, somatic mutations, and *NF1* mutations, showed no prognostic significance in patients with JMML. *PTPN11* mutation and the number of somatic alterations present at diagnosis both appeared statistical significance for EFS. HSCT could improve the outcome in JMML significantly. *P* values < 0.05% are shown in italics

Moreover, when the patients were divided into group with HSCT and group without HSCT, no relationship was found between these characteristics and disease outcome except age (*P* = 0.008) (Table 4). Patients with HSCT showed no differences in sex, platelet count, elevated HbF concentration for age, karyotype, monosomy 7 status, mutation genes, and the number of alterations in comparison with patients without HSCT (*P* > 0.05). But the HSCT group was enriched with patients older than 24 months in comparison with the no-HSCT group (*P* = 0.031).

**Table 4:** Univariate analysis of the EFS in JMML when the patients divided into HSCT and no-HSCT group

Variable	HSCT cases (*N* = 45)				no-HSCT cases (*N* = 48)			
	*N*/total	EFS (%)	HR (95% CI)	*P*	*N*/total = 48	EFS (%)	HR (95% CI)	*P*
Gender								
Female	15/45	53.3	1		17/48	42.5	1	
Male	30/45	54.3	0.84 (0.33–2.10)	0.706	31/48	26.7	1.54 (0.68–3.51)	0.287
Age at diagnosis								
≤ 24 months	19/45	83.9	1		31/48	28.5	1	
> 24 months	26/45	32.6	5.36 (1.56–18.39)	***0.008***	17/48	38.8	0.85 (0.39–1.84)	0.67
Platelet count at diagnosis (× 10^3^/μL)								
≥ 40	30/45	52.2	1		29/47	37.6	1	
< 40	15/45	59.3	1.05 (0.40–2.73)	0.926	18/47	30.9	1.47 (0.69–3.15)	0.318
Myeloid or erythroid precursors on PB smear								
No	8/43	43.8	1		9/38	29.2	1	
Yes	35/43	59.3	0.72 (0.23–2.16)	0.548	29/38	33.9	1.44 (0.49–4.27)	0.509
Fetal hemoglobin at diagnosis								
≤ 10%	5/29	80.0	1		2/16	50	1	
> 10%	24/29	52.6	2.88 (0.37–22.35)	0.311	14/16	35.8	1.58 (0.19–12.97)	0.672
Monosomy 7								
Negative	24/33	54.2	1		23/26	33.2	1	
Positive	9/33	88.9	0.22 (0.03–1.71)	0.147	3/26	0	1.58 (0.46–5.48)	0.469
Karyotype								
Normal	20/34	58.2	1		21/28	39.4	1	
Abnormal	14/34	57.1	0.96 (0.33–2.77)	0.938	7/28	0	1.70 (0.64–4.54)	0.289
*NF1* status								
Mutation absent	29/45	60.0	1		38/48	32.2	1	
Mutation present	16/45	43.8	1.65 (0.69–3.40)	0.264	10/48	43.8	0.73 (0.28–1.94)	0.532
*PTPN11* status								
Mutation absent	24/45	65.2	1		31/48	49.8	1	
Mutation present	21/45	42.9	1.72 (0.70–4.22)	0.233	17/48	6.4	3.70 (1.71–8.00)	*0.001*
Somatic alterations at diagnosis								
0 or 1	27/45	69.2	1		34/48	43.4	1	
2 or more	18/45	33.3	2.57(1.05–6.29)	***0.039***	14/48	14.3	2.07 (0.98–4.39)	0.058
Mutation subtype								
Only one non-*PTPN11* mutation	16/45	67.7	1		25/48	54.6	1	
Only one *PTPN11* mutation	11/45	72.7	0.68 (0.16–2.84)	0.595	9/48	13.3	3.77 (1.36–10.44)	*0.011*
Combined mutations without *PTPN11*	8/45	62.5	1.01 (0.24–4.22)	0.994	6/48	33.3	1.79 (0.55–5.82)	0.336
*PTPN11* mutation with additional alterations	10/45	10.0	3.60 (1.20–10.79)	***0.022***	8/48	0	4.44 (1.68–11.78)	*0.003*

*P* values < 0.05% are shown in italics

### Expanding the spectrum of RAS-pathway mutations

**Fig. 1:**
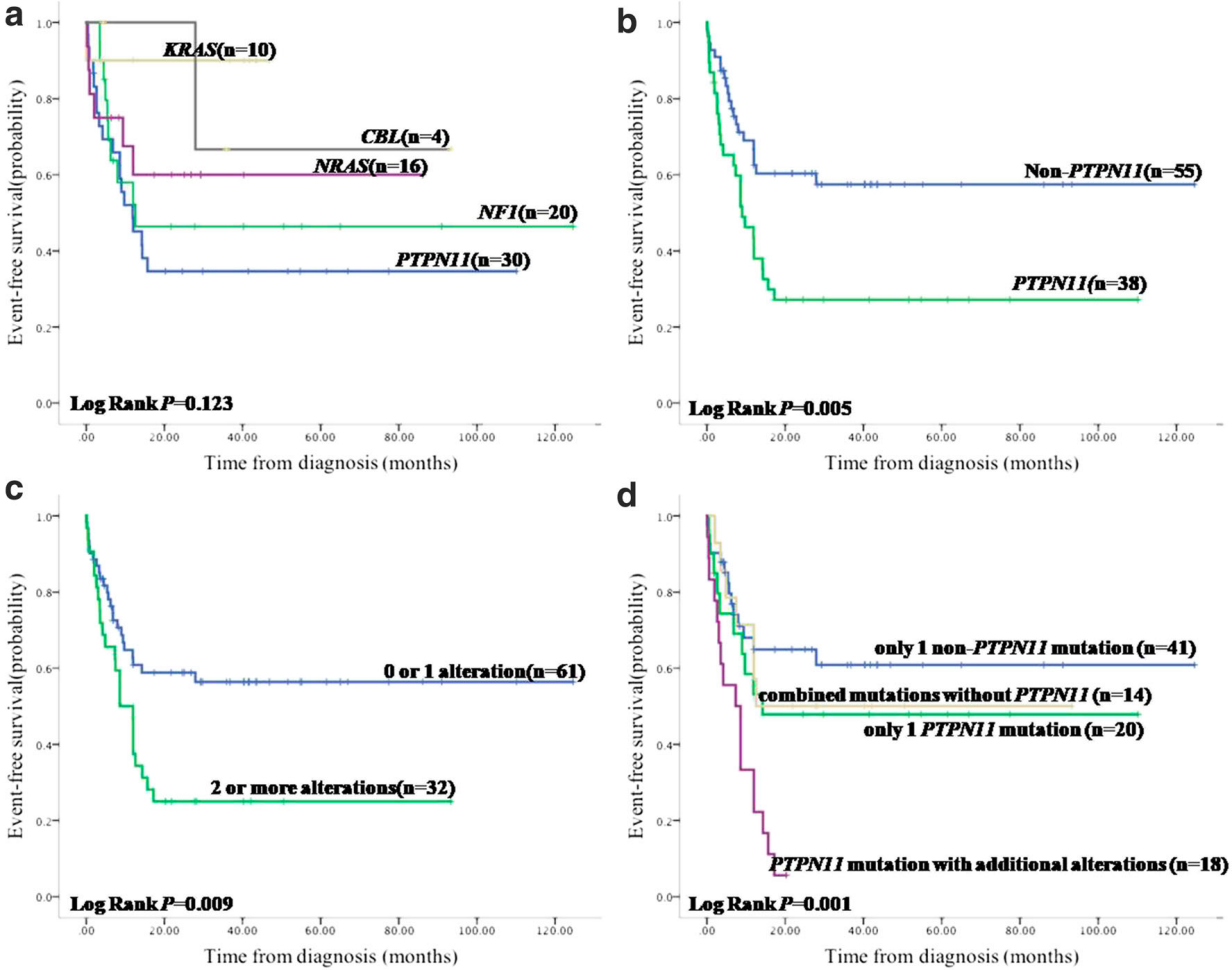
The EFS and genotype in JMML. **a** EFS based on the type of the five classical RAS-signaling genes. No significant difference was noted. **b** EFS based on status of *PTPN11*. Patients with *PTPN11* mutation exhibited significantly lower EFS compared with non-*PTPN11* mutations. **c** EFS based on the number of somatic events. Patients without or with one somatic alteration at diagnosis showed significantly better prognosis in comparison to those with more than two alterations. **d** EFS based on the four subgroups stratified by the mutational gene and number. *PTPN11* mutation with additional alteration exhibited poorer outcome when compared with other three subtypes, only one non-*PTPN11* mutation, only one *PTPN11* mutation, and combined mutations without *PTPN11*.

Although RAS-pathway lesions have traditionally been thought to represent largely mutually exclusive events [12], coexisting mutations in *NRAS*, *KRAS*, *PTPN11*, *CBL*, and *NF1* were found in 10 of 93 (10.8%) patients, 3 with *PTPN11* and *NF1*, 2 with *PTPN11* and *CBL*, 1 with *PTPN11* and *NRAS*, 1 with *NF1* and *NRAS*, 1 with *NF1* and *KRAS*, 1 with *PTPN11* and *NRAS* and *KRAS*, and 1 with *PTPN11* and *NRAS* and *CBL.* Acquisition of *NF1* haploinsufficiency with *PTPN11* was the most frequent subclone event. No significant difference in EFS was noted among the five RAS-signaling genes (*P* = 0.123) (Fig. 1a). No difference was found between germline and somatic mutation either (EFS 66.7% vs. 44.0%, hazard ratio (HR) = 1.53, confidence interval (CI) = 0.21–11.15, *P* = 0.675) (Table 3).

However, patients with *PTPN11* mutation exhibited significantly lower EFS compared with non-*PTPN11* mutations (EFS 27.2% vs. 57.4%, HR = 2.25, CI = 1.27–3.99, *P* = 0.005) (Fig. 1b). There were no differences in age, sex, distribution of clinical parameters, and HSCT treatment in patients with *PTPN11* or non-*PTPN11* mutations (*P* > 0.05).

### The secondary mutational signature correlating with disease aggressiveness

In addition to the classical JMML-associated mutations affecting RAS-pathway genes (*PTPN11*, *NF1*, *KRAS*, *NRAS*, and *CBL*), gene sequencing detected secondary mutations in *ASXL1* (7/93, 7.5%), *JAK3* (6/93, 6.4%), *SETBP1* (4/93, 4.3%), *EZH2* (2/93, 2.2%), *JAK2* (1/93, 1.1%), *SRSF2* (1/93, 1.1%), *GATA2* (1/93, 1.1%), and *NOTCH1* (1/93, 1.1%), many of which were known epigenetic modifiers including members of the polycomb repressive complex 2 (PRC2) or genes implicated in RAS-RAF-MEK-ERK pathway activation.

Five of the six patients with *JAK3* mutations at diagnosis co-occurred with *PTPN11* mutations and all six patients went on to poor outcome, uncovering activated JAK-STAT corresponding with clinical outcome.

We identified a mutation in *GATA2*, a transcription factor broadly involved in hematopoiesis [18] and a mutation in *SRSF2*, a member of the spliceosome complex. Recent work has shown that germline *GATA2* mutations are responsible for several syndromes, leading to a predisposition to myeloid malignancies [7, 9].

In contrast to previous reports that emphasized the rarity of genetic mutations in epigenetic modifier genes in JMML [8, 19, 22], we identified components of PRC2, including *EZH2* and *ASXL1*, were mutated at diagnosis (9/93, 9.68%). Mutations in epigenetic modifiers are frequent in JMML.

### Somatic alterations at diagnosis predicting outcome

Using the number of somatic events at diagnosis, we evaluated differences in prognosis. In our 93-patient cohort, patients without or only with one somatic alteration at diagnosis showed significantly better prognosis in comparison with those with more than two alterations (EFS 56.4% vs. 25.0%, HR = 2.13, CI = 1.21–3.76, *P* = 0.009) (Fig. 1c).

**Fig. 2:**
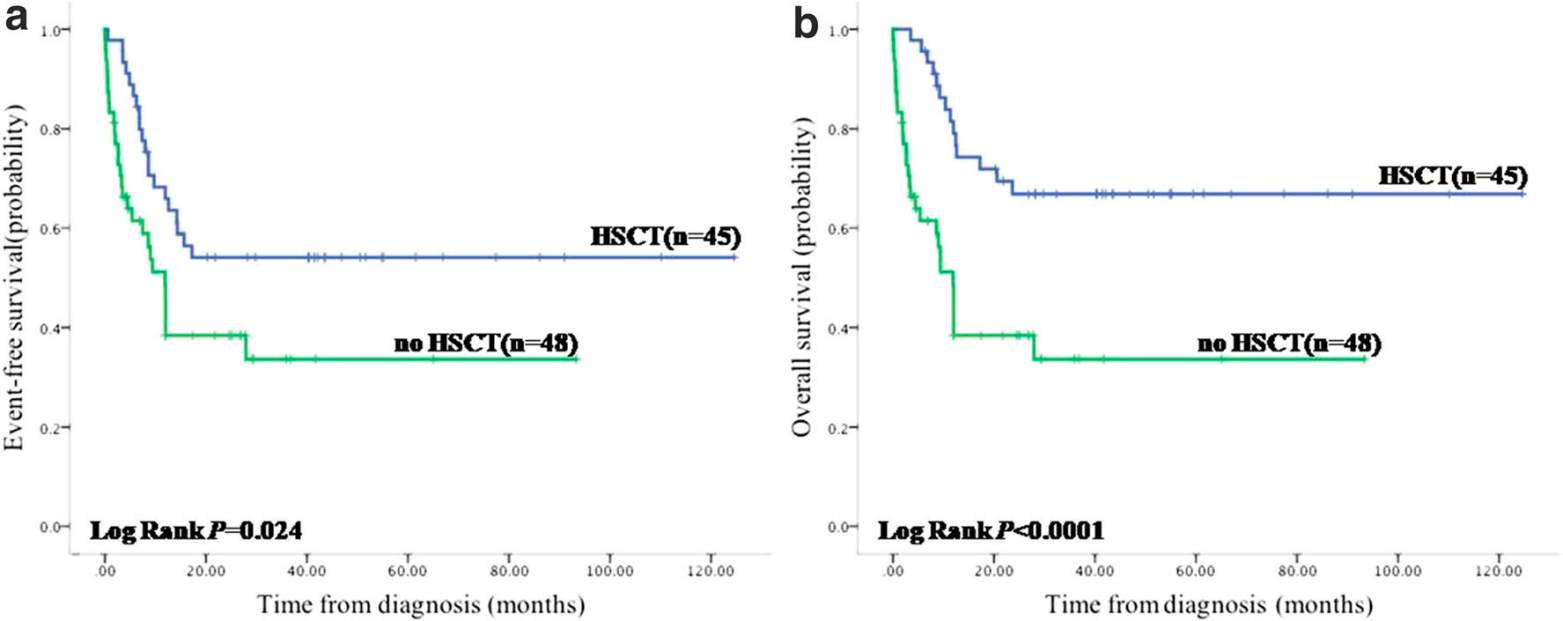
HSCT can improve the EFS and OS in JMML significantly

HSCT could improve the EFS and OS significantly (EFS 54.1% vs. 33.6%, HR = 1.95, CI = 1.09–3.47, *P* = 0.024 and OS 66.8% vs. 33.6%, HR = 3.09, CI = 1.67–5.85, *P* < 0.0001) compared with patients without HSCT (Fig. 2). Although most of patients received chemotherapy before HSCT, we thought it was not beneficial to the final outcome because intensity of chemotherapy (non-, mild, or AML-like) prior to HSCT did not induce remission and showed no impact on HSCT outcome (*P* = 0.767), being not in favor of intense chemotherapy before transplantation, to avoid the adverse reactions associated with intense chemotherapy.

The OS of no-HSCT patients in our study was better than some other reports, maybe because this group was enriched with more *KRAS* (11/43, 25.6%) and *CBL* (4/43, 9.3%) mutations, 1 germline mutation in *PTPN11*, and 1 germline mutation in *NF1*.

Furthermore, when a Cox multivariate regression model was applied, *PTPN11* mutation remained independently prognostic of poor outcome (EFS HR = 2.57, CI = 1.41–4.69, *P* = 0.002) after adjusting for the number of alterations and HSCT treatment. Two or more somatic events also remained independently prognostic of poor prognosis (EFS HR = 2.05, CI = 1.15–3.67, *P* = 0.015) after adjusting for *PTPN11* status and HSCT (Table 5). Not only *PTPN11* mutation but also the number of somatic alterations at diagnosis retained statistical significance for EFS.

**Table 5:** Multivariate analysis of the survival parameters in JMML

Variable	*N*	EFS (%)	HR (95% CI)	*P*
*PTPN11* status				
Mutation absent	55	57.4	1	
Mutation present	38	27.2	2.57 (1.41–4.69)	*0.002*
Somatic alterations at diagnosis				
0 or 1	61	56.4	1	
2 or more	32	25.0	2.05 (1.15–3.67)	*0.015*
HSCT				
Yes	45	54.1	1	
No	48	33.6	2.66 (1.46–4.84)	*0.001*
Mutation subtype				
Only one non-*PTPN11* mutation	41	60.9	1	
Only one *PTPN11* mutation	20	47.8	1.63 (0.71–3.72)	0.246
Combined mutations without *PTPN11*	14	50.0	1.32 (0.53–3.31)	0.556
*PTPN11* mutation with additional alterations	18	5.6	3.88 (1.87–8.05)	***<*** *0.0001*

When a Cox multivariate regression model was applied, *PTPN11* mutation and the number of somatic alterations remained independently prognostic of poor outcome after adjusting for the improvement of HSCT treatment. Then, the cohort was subdivided. *PTPN11* mutation with additional alteration showed the poorest outcome in comparison with those with only one non-*PTPN11* mutation, only one *PTPN11* mutation, and combined mutations without *PTPN11*. HSCT could improve the outcome significantly. *P* values < 0.05% are shown in italics

On account of the relevance between *PTPN11* status and the number of mutations (*P* = 0.029) and some patients harboring both characteristics, 93 cases were then subdivided into four groups stratified by their mutational gene and number, only one non-*PTPN11* mutation (41/93, 44.0%), only one *PTPN11* mutation (20/93, 21.5%), combined mutations but without *PTPN11* abnormity (14/93, 15.1%), and *PTPN11* mutation with additional somatic alteration (18/93, 19.4%). The *PTPN11*-mutated subgroup of patients showed the shortest survival which worsened when a secondary mutation was present (EFS 5.6%, HR = 3.73, CI = 1.83–7.62, *P* < 0.0001). The subgroup of only one *PTPN11* mutation (EFS 47.8%, HR = 1.45, CI = 0.64–3.27, *P* = 0.369) and the subgroup holding combined mutations, but excluding *PTPN11* abnormity (EFS 50.0%, HR = 1.27, CI = 0.51–3.14, *P* = 0.610), exhibited a little more hazard compared with the subtype of only one non-*PTPN11* mutation (EFS 60.9%), albeit no statistically significant difference (Fig. 1d).

## Discussion

JMML is characterized by the presence of mutations activating the RAS-signaling pathway in about 90% of cases [1, 6], in which the mutation of *PTPN11* is a marker of poor prognosis. Recent studies have shown that oncogenic RAS signaling can mediate genomic DNA methylation [6, 11, 21]. RAS-activating mutations in different genes might have distinct effects on epigenome remodeling. Analyzing 167 cases, the EWOG-MDS demonstrated that the low-methylation cluster comprised patients with *CBL* and *NRAS* mutations known to have a favorable prognosis. *KRAS* mutation was associated with the intermediate cluster. The high methylation group was dominated by cases with *PTPN11* mutation, resulting in poor outcome, suggesting RAS-pathway mutation patterns define epigenetic subclasses in JMML [10]. In addition, hinting at possible functional links between Ras activation and methylation classes, some research also reported that DNA hypermethylation was more pronounced when additional mutations in Ras-pathway genes or epigenetic modifier genes were present [10, 17].

Furthermore, we have shown that secondary mutations providing additional activation of RAS-signaling and other signaling pathways were frequent in poor-outcome JMML. It seemed that these secondary mutations, as second hits targeting the RAS pathway, contribute to further augment the extent of epigenetic remodeling. Pre-existing epigenetic alterations might provide a fertile ground for malignant transformation following single or few genetic hits. This sequence of events has been shown in lung cancer where hypermethylation of PRC2 target genes sensitizes epithelial cells to single-step transformation by mutant *KRAS* [24]. No matter whether the RAS-signaling genes or the so-called secondary mutations are primary to drive disease, it implies that each mutation in a series of mutations has biological activity, more mutations, more hits, thus maybe more aggressive.

In our cohort, DNA was extracted from PB or BM. The mutation frequencies in PB or BM were still comparable because leukemia cells could distribute in both PB and BM with almost the same proportion in JMML other than the predominant rate in BM in acute leukemia. Thus, the outcome determined by the mutation patterns was not biased regardless of sample sources.

In conclusion, both *PTPN11* mutation and the number of somatic alterations detected at diagnosis are likely to be the major determinant of outcome in JMML. Notably, in addition to identifying patients with aggressive disease, our data provides patient selection criteria for therapeutic options in this heterogeneous childhood leukemia. Yet, the effects of RAS-signaling genes on epigenome remodeling and the interactions of these secondary events in RAS-signaling and other signaling pathways remain to be explored.

## Abbreviation

AML: Acute myeloid leukemia
BM: Bone marrow
CI: Confidence interval
EFS: Effect-free survival
HbF: Hemoglobin F
HR: Hazard ratio
HSCT: Hematopoietic stem cell transplantation
JMML: Juvenile myelomonocytic leukemia
OS: Overall survival
PB: Peripheral blood
PRC2: Polycomb repressive complex 2
